# Poly[[tetra­aqua­bis­(μ_3_-pyridine-2,6-dicarboxyl­ato)(μ_2_-pyridine-2,6-dicarboxyl­ato)dilanthanum(III)] dihydrate]

**DOI:** 10.1107/S1600536811030807

**Published:** 2011-08-06

**Authors:** Shie Fu Lush, Fwu Ming Shen

**Affiliations:** a Department of General Education Center, Yuanpei University, HsinChu 30015, Taiwan; b Department of Biotechnology, Yuanpei University, No. 306 Yuanpei St, HsinChu 30015 Taiwan

## Abstract

There are two independent La^III^ cations in the polymeric title compound, {[La_2_(C_7_H_3_NO_4_)_3_(H_2_O)_4_]·2H_2_O}_*n*_. One is nine-coordinated in an LaN_2_O_7_ tricapped trigonal–prismatic geometry formed by three pyridine-2,6-dicarboxyl­ate anions and two water mol­ecules, while the other is ten-coordinated in an LaNO_9_ bicapped square-anti­prismatic geometry formed by four pyridine-2,6-dicarboxyl­ate anions and two water mol­ecules. The two La^III^ cations are separated by a non-bonding distance of 5.026 (3) Å. The pyridine-2,6-dicarboxyl­ate anions bridge the La^III^ cations, forming a three-dimensional polymeric complex. The crystal structure contains extensive classical O—H⋯O hydrogen bonds and weak inter­molecular C—H⋯O hydrogen bonds. The crystal structure is further consolidated by π–π stacking between pyridine rings, the shortest centroid–centroid distance between parallel pyridine rings being 3.700 (5) Å.

## Related literature

For applications of lanthanide metal carboxyl­ate systems in supra­molecular chemistry and functional materials, see: Yang *et al.* (2011 ▶); Chantal *et al.* (2008 ▶). For similar structures, see: Brouca *et al.* (2002 ▶); Ghosh & Bharadwaj (2004 ▶).
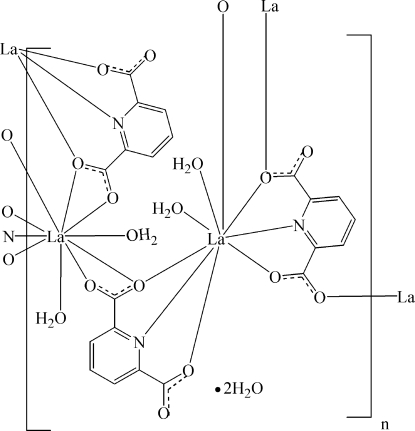


## Experimental

### 

#### Crystal data

[La_2_(C_7_H_3_NO_4_)_3_(H_2_O)_4_]·2H_2_O
*M* *_r_* = 881.23
Triclinic, 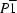

*a* = 10.4910 (2) Å
*b* = 10.9197 (2) Å
*c* = 13.0850 (3) Å
α = 77.915 (1)°
β = 76.702 (1)°
γ = 86.049 (1)°
*V* = 1426.14 (5) Å^3^
*Z* = 2
Mo *K*α radiation
μ = 3.04 mm^−1^
*T* = 293 K
0.17 × 0.13 × 0.11 mm

#### Data collection

Nonius KappaCCD diffractometer
Absorption correction: multi-scan (*SCALEPACK*; Otwinowski & Minor, 1997 ▶) *T*_min_ = 0.592, *T*_max_ = 0.699
10097 measured reflections
4496 independent reflections
4014 reflections with *I* > 2σ(*I*)
*R*_int_ = 0.059

#### Refinement

*R*[*F* ^2^ > 2σ(*F* ^2^)] = 0.045
*wR*(*F* ^2^) = 0.126
*S* = 1.03
4496 reflections
398 parameters
H-atom parameters constrained
Δρ_max_ = 3.36 e Å^−3^
Δρ_min_ = −1.06 e Å^−3^

### 

Data collection: *COLLECT* (Nonius, 2000 ▶); cell refinement: *SCALEPACK* (Otwinowski & Minor, 1997 ▶); data reduction: *DENZO* (Otwinowski & Minor, 1997 ▶) and *SCALEPACK*; program(s) used to solve structure: *SHELXS97* (Sheldrick, 2008 ▶); program(s) used to refine structure: *SHELXL97* (Sheldrick, 2008 ▶); molecular graphics: *PLATON* (Spek, 2009 ▶); software used to prepare material for publication: *PLATON*.

## Supplementary Material

Crystal structure: contains datablock(s) global, I. DOI: 10.1107/S1600536811030807/xu5269sup1.cif

Structure factors: contains datablock(s) I. DOI: 10.1107/S1600536811030807/xu5269Isup2.hkl

Additional supplementary materials:  crystallographic information; 3D view; checkCIF report

## Figures and Tables

**Table 1:** Selected bond lengths (Å)

La1—N1	2.644 (5)
La1—N2^i^	2.728 (6)
La1—O1	2.502 (5)
La1—O3	2.614 (5)
La1—O8^i^	2.575 (5)
La1—O11^i^	2.578 (5)
La1—O11^ii^	2.600 (5)
La1—O13	2.593 (5)
La1—O14	2.525 (5)
La2—N3^iii^	2.688 (6)
La2—O3	2.674 (5)
La2—O4	2.605 (5)
La2—O5	2.865 (6)
La2—O6	2.591 (5)
La2—O6^iii^	2.615 (5)
La2—O7	2.524 (5)
La2—O10^iii^	2.539 (5)
La2—O15	2.574 (5)
La2—O16	2.575 (6)

Symmetry codes: (i) 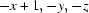; (ii) 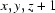; (iii) 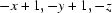.

**Table 2:** Hydrogen-bond geometry (Å, °)

*D*—H⋯*A*	*D*—H	H⋯*A*	*D*⋯*A*	*D*—H⋯*A*
O13—H13*A*⋯O5	0.85	2.04	2.766 (8)	143
O13—H13*B*⋯O1^iv^	0.85	1.91	2.721 (7)	160
O14—H14*A*⋯O5	0.97	2.23	3.062 (8)	143
O14—H14*B*⋯O9^v^	0.83	2.44	3.225 (10)	160
O14—H14*B*⋯O10^v^	0.83	2.22	2.712 (8)	119
O15—H15*A*⋯O7^iii^	0.85	2.14	2.948 (8)	160
O15—H15*B*⋯O12^ii^	0.85	2.16	2.849 (7)	138
O16—H16*A*⋯O8	0.85	2.12	2.760 (8)	132
O16—H16*B*⋯O17^iii^	0.85	2.05	2.843 (15)	155
O17—H17*A*⋯O9^vi^	0.82	2.13	2.816 (17)	141
O17—H17*B*⋯O9^vii^	0.82	2.39	2.758 (15)	108
O18—H18*A*⋯O2^viii^	0.82	2.31	2.75 (2)	114
O18—H18*B*⋯O18^ix^	0.88	2.46	2.89 (3)	110
C11—H11⋯O2^x^	0.93	2.45	3.320 (11)	155
C12—H12⋯O18^xi^	0.93	2.51	3.36 (2)	152

Symmetry codes: (ii) 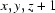; (iii) 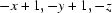; (iv) 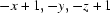; (v) 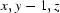; (vi) 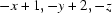; (vii) 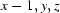; (viii) 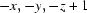; (ix) 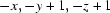; (x) 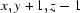; (xi) 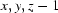.

**Fig. 1.**
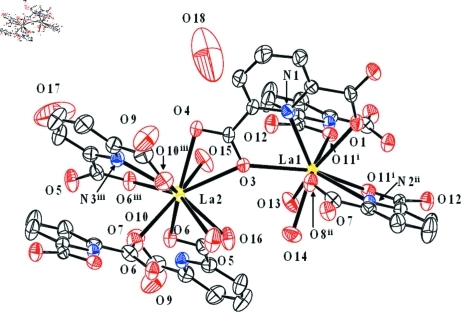
View of the title compound with the atom numbering scheme. Displacement ellipsoids for non-H atoms are drawn at the 50% probability level. H atoms have been omitted for clarity [symmetry code:(i) x, y, z + 1; (ii) -x + 1, -y, -z; (iii) -x + 1, -y + 1, -z].

**Fig. 2.**
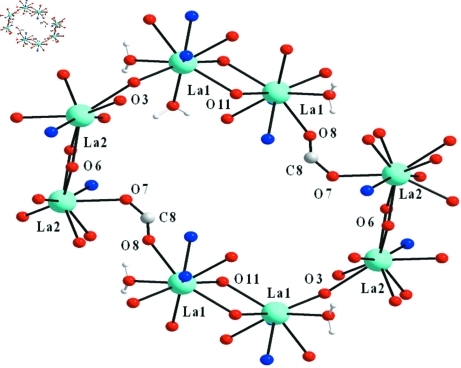
Perspective view of carboxylate-bridged-metal rings of La8C2O10.

**Fig. 3.**
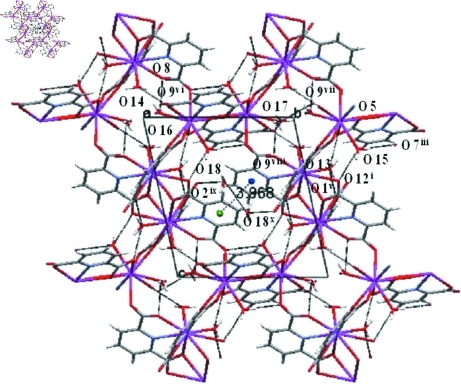
The molecular packing for the title compound, viewed along the a axis. Hydrogen bonds are shown as dashed lines.

## supplementary crystallographic information

### Comment

Pyridine-2,6-dicarboxylic acid (pydH_2_) and its deprotonated anion behave as multifunctional ligands to act as bridging ligands in metal complexes with five coordination sites involving the oxygen atoms of the carboxylate groups and the nitrogen atom of the pyridine ring. In recent years, the chemistry of lanthanide metal carboxylate systems is of great interest because of their extensive usage in supramolecular chemistry and functional materials (Brouca *et al.* 2002; Ghosh *et al.* 2004; Yang *et al.* 2011; Chantal *et al.* 2008). Here, we report a new La^III^ complex with pyridine-2,6-dicarboxylic acid, [[La_2_(pyd)_3_(H_2_O)_4_].2H_2_O]_n_, from hydrothermal reaction.

The structure of the title compound is shown as Fig 1. There are two independent La^III^ ions where La(1) is nine coordinated with N_2_O_7_ donors sets to form tricapped trigonal prism geometries, where La(2) is ten coordinated with NO_9_ donor sets to from bicapped square antiprisms geometries. The selected bond lengths (Å) of title compound are listed in Table 2. The La^III^—O and La^III^—N distances are similar to those found in other La^III^ complex (Brouca, *et al.*2002; Ghosh, *et al.* 2004). The bond distances and bond angles in the ligand moiety are within normal ranges.

The structure consists of two types of ligand-binding modes contributing to link the LaO_5_N_2_(H_2_O)_2_ and LaO_7_N(H_2_O)_2_ polyhedral chains to three-dimensional network. This network can be described in terms of a 20-membered ring related to each other by the intermediate C8 carboxylate group. It results in a La1—La1 distance equal to 4.440 (1) Å. A projection of one 20-membered ring along *x* axis is shown in Fig. 2. Rings built from eight lanthanum atoms can be seen. In these rings, long La—La distances are found:La1—La2 = 6.190 (3) Å through C8 carboxylate group of pda1, La1—La2 =5.026 (3) Å through C7 carboxylate group of pda2. The La2—La2 distance through theµ-O6 atom is apart from the others with the shortest value of 4.514 (2) Å.

In the title crystal structure stabilized *via* O—H···O and weak C—H···O hydrogen bonds (Fig.3) (full details and symmetry codes are given in Table 3). The π–π stacking interaction is also observed, the centroid···centroid distance between the parallel aryl ring being 3.969 (4)Å and 3.700 (5) Å [*Cg*2^vi^···*Cg*2(N2/C9—C13), *Cg*3^vii^···*Cg*3(N3/C16—C20)] (symmetry code: (vi)1-*X*, 1-Y, 1-*Z*,(vii) 2-*X*, 1-Y, –*Z*). C1—O2···*Cg*1^viii^((N1/C2—C6) is 3.892 (6) Å((viii) –*X*, –Y, 1-*Z*).

### Experimental

LaCl_3_.6H_2_O (0.0899 g, 0.25 mmol), pydridine-2,6-dicarboxylic acid (0.0418 g, 0.25 mmol) and 1,2-bis(4-pyridyl)ethane were mixed in 10 ml of deionized water. After stirring for 30 min, the mixture was placed in a 23 ml Teflon-lined reactor, heated at 453 K for 72 h, then cooled slowly to room temperature. The colorless transparent single crystals of the title compound were obtained in 35.10% yield (based on La).

### Refinement

Water H atoms were placed in chemical sensible positions and *U*_iso_(H)= 1.5*U*_eq_(O). Other H atoms were positioned geometrically with C—H = 0.93 Å, and refined using a riding model with *U*_iso_(H) = 1.2*U*_eq_(C). The precise of the structure is low.

### Crystal data

**Table:**

[La_2_(C_7_H_3_NO_4_)_3_(H_2_O)_4_]·2H_2_O	*Z* = 2
*M**_r_* = 881.23	*F*(000) = 852
Triclinic, *P*1	*D*_x_ = 2.052 Mg m^−^^3^
Hall symbol: -P 1	Mo *K*α radiation, λ = 0.71073 Å
*a* = 10.4910 (2) Å	Cell parameters from 11712 reflections
*b* = 10.9197 (2) Å	θ = 2.0–25.4°
*c* = 13.0850 (3) Å	µ = 3.04 mm^−^^1^
α = 77.915 (1)°	*T* = 293 K
β = 76.702 (1)°	Prism, colorless
γ = 86.049 (1)°	0.17 × 0.13 × 0.11 mm
*V* = 1426.14 (5) Å^3^	

### Data collection

**Table:**

Nonius KappaCCD diffractometer	4496 independent reflections
Radiation source: fine-focus sealed tube	4014 reflections with *I* > 2σ(*I*)
graphite	*R*_int_ = 0.059
Detector resolution: 9 pixels mm^-1^	θ_max_ = 24.2°, θ_min_ = 2.8°
ω/2θ scans	*h* = −12→11
Absorption correction: multi-scan (*SCALEPACK*; Otwinowski & Minor, 1997)	*k* = −12→12
*T*_min_ = 0.592, *T*_max_ = 0.699	*l* = −14→15
10097 measured reflections	

### Refinement

**Table:**

Refinement on *F*^2^	Secondary atom site location: difference Fourier map
Least-squares matrix: full	Hydrogen site location: inferred from neighbouring sites
*R*[*F*^2^ > 2σ(*F*^2^)] = 0.045	H-atom parameters constrained
*wR*(*F*^2^) = 0.126	*w* = 1/[σ^2^(*F*_o_^2^) + (0.0734*P*)^2^ + 6.7391*P*] where *P* = (*F*_o_^2^ + 2*F*_c_^2^)/3
*S* = 1.03	(Δ/σ)_max_ = 0.001
4496 reflections	Δρ_max_ = 3.36 e Å^−^^3^
398 parameters	Δρ_min_ = −1.06 e Å^−^^3^
0 restraints	Extinction correction: *SHELXL97* (Sheldrick, 2008), Fc*=KFc[1+0.001Fc^2^λ^3^/sin(2θ)]^-1/4^
Primary atom site location: structure-invariant direct methods	Extinction coefficient: 0.0024 (7)

### Special details

**Table:**

Geometry. Bond distances, angles *etc*. have been calculated using the rounded fractional coordinates. All su's are estimated from the variances of the (full) variance-covariance matrix. The cell e.s.d.'s are taken into account in the estimation of distances, angles and torsion angles
Refinement. Refinement on *F*^2^ for ALL reflections except those flagged by the user for potential systematic errors. Weighted *R*-factors *wR* and all goodnesses of fit *S* are based on *F*^2^, conventional *R*-factors *R* are based on *F*, with *F* set to zero for negative *F*^2^. The observed criterion of *F*^2^ > σ(*F*^2^) is used only for calculating -*R*-factor-obs *etc*. and is not relevant to the choice of reflections for refinement. *R*-factors based on *F*^2^ are statistically about twice as large as those based on *F*, and *R*-factors based on ALL data will be even larger.

### Fractional atomic coordinates and isotropic or equivalent isotropic displacement parameters (Å^2^)

**Table:**

	*x*	*y*	*z*	*U*_iso_*/*U*_eq_	
La1	0.44785 (3)	−0.02327 (3)	0.35019 (3)	0.0209 (2)	
La2	0.41829 (4)	0.30745 (3)	0.02503 (3)	0.0252 (2)	
O1	0.3054 (5)	−0.1677 (5)	0.5004 (4)	0.0337 (17)	
O2	0.1185 (5)	−0.2630 (5)	0.5862 (5)	0.0492 (19)	
O3	0.3593 (4)	0.1173 (4)	0.1926 (4)	0.0272 (16)	
O4	0.1922 (5)	0.2287 (5)	0.1428 (4)	0.0399 (17)	
O5	0.6510 (6)	0.2726 (5)	0.1081 (5)	0.0463 (19)	
O6	0.6152 (5)	0.4494 (4)	0.0013 (4)	0.0328 (17)	
O7	0.5246 (5)	0.3575 (5)	−0.1722 (4)	0.0356 (17)	
O8	0.5833 (5)	0.1871 (4)	−0.2427 (4)	0.0319 (17)	
O9	0.9032 (7)	0.8529 (6)	0.0852 (8)	0.081 (3)	
O10	0.7051 (5)	0.8128 (5)	0.0666 (4)	0.0393 (17)	
O11	0.3996 (4)	0.0804 (4)	−0.4828 (4)	0.0262 (14)	
O12	0.3094 (5)	0.2114 (5)	−0.6006 (4)	0.0374 (17)	
O13	0.5983 (5)	0.1647 (5)	0.3240 (4)	0.0423 (17)	
O14	0.6366 (5)	−0.0113 (5)	0.1893 (4)	0.0446 (17)	
O15	0.3833 (7)	0.3857 (5)	0.2021 (4)	0.051 (2)	
O16	0.5634 (7)	0.1192 (5)	−0.0243 (5)	0.053 (2)	
N1	0.1958 (5)	−0.0293 (5)	0.3519 (4)	0.0275 (17)	
N2	0.4329 (5)	0.2539 (5)	−0.3812 (4)	0.0236 (17)	
N3	0.7679 (6)	0.5744 (5)	0.0726 (5)	0.0294 (17)	
C1	0.1846 (7)	−0.1889 (7)	0.5121 (6)	0.032 (2)	
C2	0.1188 (7)	−0.1139 (6)	0.4260 (6)	0.031 (2)	
C3	−0.0104 (8)	−0.1294 (8)	0.4229 (7)	0.049 (3)	
C4	−0.0596 (8)	−0.0556 (10)	0.3409 (8)	0.061 (3)	
C5	0.0167 (8)	0.0337 (9)	0.2682 (7)	0.048 (3)	
C6	0.1450 (7)	0.0440 (7)	0.2754 (6)	0.029 (2)	
C7	0.2362 (7)	0.1378 (6)	0.1992 (5)	0.028 (2)	
C8	0.5249 (7)	0.2914 (7)	−0.2401 (6)	0.031 (2)	
C9	0.4483 (7)	0.3385 (7)	−0.3250 (6)	0.029 (2)	
C10	0.3945 (8)	0.4592 (7)	−0.3418 (6)	0.041 (3)	
C11	0.3248 (10)	0.4928 (8)	−0.4212 (8)	0.057 (3)	
C12	0.3085 (9)	0.4063 (7)	−0.4800 (7)	0.045 (3)	
C13	0.3647 (7)	0.2886 (7)	−0.4586 (6)	0.029 (2)	
C14	0.3545 (6)	0.1889 (6)	−0.5189 (5)	0.0235 (19)	
C15	0.6790 (7)	0.3836 (6)	0.0665 (5)	0.031 (2)	
C16	0.7822 (7)	0.4492 (7)	0.0935 (6)	0.034 (2)	
C17	0.8816 (8)	0.3878 (8)	0.1402 (7)	0.047 (3)	
C18	0.9714 (9)	0.4597 (9)	0.1617 (9)	0.057 (3)	
C19	0.9565 (8)	0.5869 (8)	0.1416 (8)	0.049 (3)	
C20	0.8519 (8)	0.6423 (7)	0.0988 (6)	0.037 (2)	
C21	0.8212 (8)	0.7801 (8)	0.0811 (7)	0.043 (3)	
O17	0.1654 (11)	0.9097 (12)	0.0235 (15)	0.203 (8)	
O18	0.0497 (18)	0.4499 (17)	0.4043 (14)	0.262 (11)	
H3	−0.06240	−0.18810	0.47480	0.0590*	
H4	−0.14450	−0.06660	0.33500	0.0730*	
H5	−0.01690	0.08680	0.21480	0.0570*	
H10	0.40520	0.51580	−0.30040	0.0490*	
H11	0.28900	0.57330	−0.43500	0.0680*	
H12	0.26060	0.42700	−0.53310	0.0530*	
H13A	0.60750	0.22650	0.27140	0.0510*	
H13B	0.63440	0.18290	0.37070	0.0510*	
H14A	0.67250	0.07070	0.18120	0.0670*	
H14B	0.70090	−0.05880	0.17820	0.0670*	
H15A	0.38940	0.46430	0.19540	0.0620*	
H15B	0.32620	0.35990	0.25900	0.0620*	
H16A	0.55550	0.09510	−0.08010	0.0640*	
H16B	0.64370	0.13440	−0.03160	0.0640*	
H17	0.88720	0.30080	0.15640	0.0560*	
H18	1.04150	0.42180	0.18960	0.0690*	
H19	1.01600	0.63630	0.15650	0.0590*	
H17A	0.15910	0.98620	0.01620	0.3010*	
H17B	0.12280	0.88870	−0.01490	0.3010*	
H18A	0.00000	0.44490	0.36500	0.3970*	
H18B	0.01520	0.40770	0.46880	0.3970*	

### Atomic displacement parameters (Å^2^)

**Table:**

	*U*^11^	*U*^22^	*U*^33^	*U*^12^	*U*^13^	*U*^23^
La1	0.0260 (3)	0.0196 (2)	0.0178 (3)	0.0006 (2)	−0.0064 (2)	−0.0041 (2)
La2	0.0369 (3)	0.0184 (3)	0.0199 (3)	−0.0007 (2)	−0.0063 (2)	−0.0032 (2)
O1	0.033 (3)	0.031 (3)	0.037 (3)	−0.008 (2)	−0.018 (2)	0.007 (2)
O2	0.040 (3)	0.046 (3)	0.047 (4)	−0.008 (3)	−0.002 (3)	0.017 (3)
O3	0.027 (2)	0.028 (3)	0.026 (3)	0.001 (2)	−0.0075 (19)	−0.003 (2)
O4	0.037 (3)	0.037 (3)	0.037 (3)	0.010 (2)	−0.008 (2)	0.008 (2)
O5	0.074 (4)	0.021 (3)	0.044 (3)	−0.005 (3)	−0.021 (3)	0.003 (2)
O6	0.046 (3)	0.024 (3)	0.031 (3)	−0.001 (2)	−0.017 (2)	−0.002 (2)
O7	0.051 (3)	0.033 (3)	0.028 (3)	−0.004 (2)	−0.012 (2)	−0.013 (2)
O8	0.046 (3)	0.028 (3)	0.028 (3)	0.006 (2)	−0.018 (2)	−0.011 (2)
O9	0.060 (4)	0.046 (4)	0.148 (8)	−0.008 (3)	−0.043 (5)	−0.022 (4)
O10	0.044 (3)	0.030 (3)	0.049 (3)	−0.001 (2)	−0.012 (3)	−0.017 (2)
O11	0.029 (2)	0.029 (3)	0.022 (2)	−0.001 (2)	−0.005 (2)	−0.009 (2)
O12	0.046 (3)	0.040 (3)	0.030 (3)	0.003 (2)	−0.020 (2)	−0.004 (2)
O13	0.058 (3)	0.047 (3)	0.024 (3)	−0.025 (3)	−0.012 (2)	−0.001 (2)
O14	0.049 (3)	0.041 (3)	0.037 (3)	−0.001 (3)	0.008 (3)	−0.012 (3)
O15	0.097 (5)	0.028 (3)	0.026 (3)	−0.013 (3)	−0.002 (3)	−0.007 (2)
O16	0.087 (5)	0.043 (3)	0.040 (3)	0.023 (3)	−0.030 (3)	−0.021 (3)
N1	0.029 (3)	0.026 (3)	0.026 (3)	−0.001 (2)	−0.004 (2)	−0.004 (2)
N2	0.030 (3)	0.021 (3)	0.021 (3)	0.001 (2)	−0.007 (2)	−0.006 (2)
N3	0.038 (3)	0.024 (3)	0.027 (3)	0.005 (3)	−0.009 (3)	−0.007 (3)
C1	0.044 (4)	0.023 (4)	0.024 (4)	0.004 (3)	−0.004 (3)	−0.001 (3)
C2	0.026 (3)	0.027 (4)	0.035 (4)	−0.001 (3)	−0.003 (3)	0.000 (3)
C3	0.035 (4)	0.046 (5)	0.057 (6)	−0.005 (4)	−0.012 (4)	0.013 (4)
C4	0.022 (4)	0.073 (6)	0.078 (7)	−0.013 (4)	−0.020 (4)	0.022 (5)
C5	0.035 (4)	0.054 (5)	0.048 (5)	0.006 (4)	−0.015 (4)	0.010 (4)
C6	0.029 (4)	0.025 (4)	0.032 (4)	0.006 (3)	−0.009 (3)	−0.001 (3)
C7	0.035 (4)	0.025 (4)	0.024 (4)	0.004 (3)	−0.011 (3)	−0.002 (3)
C8	0.041 (4)	0.028 (4)	0.025 (4)	−0.010 (3)	−0.003 (3)	−0.011 (3)
C9	0.032 (4)	0.028 (4)	0.027 (4)	0.001 (3)	−0.001 (3)	−0.010 (3)
C10	0.061 (5)	0.025 (4)	0.037 (4)	0.007 (4)	−0.012 (4)	−0.010 (3)
C11	0.092 (7)	0.025 (4)	0.060 (6)	0.025 (4)	−0.033 (5)	−0.016 (4)
C12	0.060 (5)	0.034 (4)	0.043 (5)	0.016 (4)	−0.024 (4)	−0.005 (4)
C13	0.032 (4)	0.030 (4)	0.025 (4)	0.004 (3)	−0.007 (3)	−0.008 (3)
C14	0.023 (3)	0.026 (4)	0.021 (3)	0.002 (3)	−0.006 (3)	−0.003 (3)
C15	0.045 (4)	0.024 (4)	0.021 (4)	−0.001 (3)	−0.003 (3)	−0.006 (3)
C16	0.036 (4)	0.034 (4)	0.032 (4)	0.005 (3)	−0.006 (3)	−0.008 (3)
C17	0.057 (5)	0.030 (4)	0.055 (5)	0.011 (4)	−0.024 (4)	−0.004 (4)
C18	0.051 (5)	0.047 (5)	0.083 (7)	0.014 (4)	−0.038 (5)	−0.014 (5)
C19	0.044 (5)	0.047 (5)	0.064 (6)	0.001 (4)	−0.022 (4)	−0.016 (4)
C20	0.041 (4)	0.039 (4)	0.035 (4)	0.003 (3)	−0.011 (3)	−0.012 (4)
C21	0.041 (4)	0.039 (4)	0.053 (5)	−0.009 (4)	−0.010 (4)	−0.014 (4)
O17	0.079 (7)	0.110 (9)	0.37 (2)	0.004 (6)	0.030 (10)	−0.030 (12)
O18	0.26 (2)	0.29 (2)	0.244 (18)	−0.205 (18)	−0.175 (17)	0.126 (17)

### Geometric parameters (Å, °)

**Table:**

La1—N1	2.644 (5)	O16—H16B	0.8500
La1—N2^i^	2.728 (6)	O17—H17A	0.8200
La1—O1	2.502 (5)	O17—H17B	0.8200
La1—O3	2.614 (5)	O18—H18A	0.8200
La1—O8^i^	2.575 (5)	O18—H18B	0.8800
La1—O11^i^	2.578 (5)	N1—C2	1.347 (9)
La1—O11^ii^	2.600 (5)	N1—C6	1.333 (9)
La1—O13	2.593 (5)	N2—C13	1.348 (9)
La1—O14	2.525 (5)	N2—C9	1.334 (9)
La2—N3^iii^	2.688 (6)	N3—C16	1.342 (10)
La2—O3	2.674 (5)	N3—C20	1.334 (10)
La2—O4	2.605 (5)	C1—C2	1.520 (11)
La2—O5	2.865 (6)	C2—C3	1.388 (12)
La2—O6	2.591 (5)	C3—C4	1.379 (13)
La2—O6^iii^	2.615 (5)	C4—C5	1.366 (14)
La2—O7	2.524 (5)	C5—C6	1.385 (12)
La2—O10^iii^	2.539 (5)	C6—C7	1.492 (10)
La2—O15	2.574 (5)	C8—C9	1.503 (11)
La2—O16	2.575 (6)	C9—C10	1.392 (11)
O1—C1	1.273 (9)	C10—C11	1.379 (13)
O2—C1	1.231 (10)	C11—C12	1.379 (13)
O3—C7	1.282 (9)	C12—C13	1.378 (11)
O4—C7	1.237 (9)	C13—C14	1.495 (10)
O5—C15	1.245 (9)	C15—C16	1.483 (11)
O6—C15	1.277 (9)	C16—C17	1.391 (12)
O7—C8	1.256 (9)	C17—C18	1.378 (13)
O8—C8	1.259 (9)	C18—C19	1.363 (14)
O9—C21	1.228 (11)	C19—C20	1.390 (12)
O10—C21	1.290 (10)	C20—C21	1.498 (12)
O11—C14	1.281 (8)	C3—H3	0.9300
O12—C14	1.239 (8)	C4—H4	0.9300
O13—H13B	0.8500	C5—H5	0.9300
O13—H13A	0.8500	C10—H10	0.9300
O14—H14A	0.9700	C11—H11	0.9300
O14—H14B	0.8300	C12—H12	0.9300
O15—H15B	0.8500	C17—H17	0.9300
O15—H15A	0.8500	C18—H18	0.9300
O16—H16A	0.8500	C19—H19	0.9300
			
O1—La1—O3	122.79 (16)	La1^i^—O8—C8	127.8 (5)
O1—La1—O13	138.55 (16)	La2^iii^—O10—C21	122.7 (5)
O1—La1—O14	144.85 (18)	La1^iv^—O11—C14	102.4 (4)
O1—La1—N1	61.51 (17)	La1^i^—O11—C14	128.0 (4)
O1—La1—O11^ii^	72.38 (16)	La1^iv^—O11—La1^i^	118.03 (17)
O1—La1—O8^i^	82.61 (17)	H13A—O13—H13B	108.00
O1—La1—O11^i^	79.37 (16)	La1—O13—H13A	125.00
O1—La1—N2^i^	70.50 (17)	La1—O13—H13B	126.00
O3—La1—O13	83.61 (15)	H14A—O14—H14B	105.00
O3—La1—O14	75.24 (16)	La1—O14—H14A	104.00
O3—La1—N1	61.30 (15)	La1—O14—H14B	130.00
O3—La1—O11^ii^	111.02 (14)	H15A—O15—H15B	108.00
O3—La1—O8^i^	78.74 (15)	La2—O15—H15A	115.00
O3—La1—O11^i^	155.18 (14)	La2—O15—H15B	126.00
O3—La1—N2^i^	134.90 (15)	H16A—O16—H16B	108.00
O13—La1—O14	66.84 (17)	La2—O16—H16A	117.00
O13—La1—N1	130.64 (17)	La2—O16—H16B	110.00
O11^ii^—La1—O13	68.07 (15)	H17A—O17—H17B	108.00
O8^i^—La1—O13	137.30 (16)	H18A—O18—H18B	108.00
O11^i^—La1—O13	71.66 (15)	La1—N1—C6	121.0 (4)
O13—La1—N2^i^	115.33 (17)	C2—N1—C6	118.9 (6)
O14—La1—N1	126.86 (16)	La1—N1—C2	120.0 (4)
O11^ii^—La1—O14	133.32 (16)	La1^i^—N2—C13	121.0 (4)
O8^i^—La1—O14	71.15 (17)	La1^i^—N2—C9	120.7 (4)
O11^i^—La1—O14	92.63 (16)	C9—N2—C13	118.1 (6)
O14—La1—N2^i^	76.04 (16)	La2^iii^—N3—C20	119.1 (5)
O11^ii^—La1—N1	92.06 (15)	C16—N3—C20	118.4 (7)
O8^i^—La1—N1	71.66 (17)	La2^iii^—N3—C16	122.5 (5)
O11^i^—La1—N1	138.66 (15)	O2—C1—C2	118.6 (7)
N1—La1—N2^i^	114.03 (17)	O1—C1—O2	125.8 (7)
O8^i^—La1—O11^ii^	154.60 (15)	O1—C1—C2	115.7 (6)
O11^ii^—La1—O11^i^	61.97 (14)	N1—C2—C1	114.6 (6)
O11^ii^—La1—N2^i^	114.01 (15)	N1—C2—C3	121.8 (7)
O8^i^—La1—O11^i^	118.28 (15)	C1—C2—C3	123.5 (7)
O8^i^—La1—N2^i^	59.34 (16)	C2—C3—C4	118.3 (8)
O11^i^—La1—N2^i^	58.97 (15)	C3—C4—C5	119.9 (8)
O3—La2—O4	49.45 (15)	C4—C5—C6	118.9 (8)
O3—La2—O5	76.72 (15)	C5—C6—C7	122.8 (7)
O3—La2—O6	121.38 (15)	N1—C6—C5	122.1 (7)
O3—La2—O7	142.63 (16)	N1—C6—C7	115.1 (6)
O3—La2—O15	69.42 (16)	O3—C7—O4	122.5 (6)
O3—La2—O16	73.31 (17)	O4—C7—C6	120.0 (7)
O3—La2—O6^iii^	134.20 (15)	O3—C7—C6	117.5 (6)
O3—La2—O10^iii^	85.13 (15)	O8—C8—C9	116.5 (7)
O3—La2—N3^iii^	121.02 (16)	O7—C8—O8	125.4 (7)
O4—La2—O5	121.66 (17)	O7—C8—C9	118.1 (7)
O4—La2—O6	146.54 (16)	N2—C9—C10	122.6 (7)
O4—La2—O7	136.93 (17)	N2—C9—C8	114.5 (6)
O4—La2—O15	72.17 (19)	C8—C9—C10	122.9 (7)
O4—La2—O16	109.57 (19)	C9—C10—C11	118.4 (7)
O4—La2—O6^iii^	101.96 (17)	C10—C11—C12	119.6 (8)
O4—La2—O10^iii^	66.56 (16)	C11—C12—C13	118.7 (8)
O4—La2—N3^iii^	72.45 (18)	C12—C13—C14	122.8 (7)
O5—La2—O6	47.20 (16)	N2—C13—C14	114.6 (6)
O5—La2—O7	98.49 (18)	N2—C13—C12	122.7 (7)
O5—La2—O15	68.8 (2)	O11—C14—C13	115.7 (6)
O5—La2—O16	65.89 (19)	O11—C14—O12	122.5 (6)
O5—La2—O6^iii^	101.06 (16)	O12—C14—C13	121.8 (6)
O5—La2—O10^iii^	137.38 (17)	O5—C15—O6	121.2 (7)
O5—La2—N3^iii^	159.07 (17)	O5—C15—C16	122.6 (7)
O6—La2—O7	72.46 (17)	O6—C15—C16	116.1 (6)
O6—La2—O15	74.76 (19)	C15—C16—C17	123.7 (7)
O6—La2—O16	93.97 (19)	N3—C16—C17	122.7 (7)
O6—La2—O6^iii^	59.73 (16)	N3—C16—C15	113.5 (6)
O6—La2—O10^iii^	145.86 (16)	C16—C17—C18	118.1 (8)
O6—La2—N3^iii^	112.19 (17)	C17—C18—C19	119.4 (9)
O7—La2—O15	143.95 (18)	C18—C19—C20	119.7 (8)
O7—La2—O16	71.09 (19)	C19—C20—C21	123.9 (8)
O6^iii^—La2—O7	83.17 (17)	N3—C20—C21	114.5 (7)
O7—La2—O10^iii^	73.54 (17)	N3—C20—C19	121.7 (7)
O7—La2—N3^iii^	74.28 (18)	O9—C21—C20	119.5 (8)
O15—La2—O16	126.2 (2)	O9—C21—O10	124.6 (8)
O6^iii^—La2—O15	67.46 (16)	O10—C21—C20	115.8 (7)
O10^iii^—La2—O15	138.7 (2)	C2—C3—H3	121.00
O15—La2—N3^iii^	105.3 (2)	C4—C3—H3	121.00
O6^iii^—La2—O16	148.18 (19)	C3—C4—H4	120.00
O10^iii^—La2—O16	72.1 (2)	C5—C4—H4	120.00
O16—La2—N3^iii^	127.1 (2)	C4—C5—H5	121.00
O6^iii^—La2—O10^iii^	118.78 (17)	C6—C5—H5	120.00
O6^iii^—La2—N3^iii^	59.01 (17)	C11—C10—H10	121.00
O10^iii^—La2—N3^iii^	60.36 (17)	C9—C10—H10	121.00
La1—O1—C1	127.8 (5)	C10—C11—H11	120.00
La1—O3—La2	143.82 (18)	C12—C11—H11	120.00
La1—O3—C7	121.3 (4)	C11—C12—H12	121.00
La2—O3—C7	91.7 (4)	C13—C12—H12	121.00
La2—O4—C7	96.0 (4)	C18—C17—H17	121.00
La2—O5—C15	89.2 (5)	C16—C17—H17	121.00
La2—O6—C15	101.4 (4)	C17—C18—H18	120.00
La2—O6—La2^iii^	120.3 (2)	C19—C18—H18	120.00
La2^iii^—O6—C15	124.5 (4)	C20—C19—H19	120.00
La2—O7—C8	126.9 (5)	C18—C19—H19	120.00
			
O3—La1—O1—C1	2.6 (7)	N3^iii^—La2—O7—C8	−87.9 (6)
O13—La1—O1—C1	124.6 (6)	O3—La2—O6^iii^—La2^iii^	105.9 (2)
O14—La1—O1—C1	−110.3 (6)	O3—La2—O6^iii^—C15^iii^	−121.3 (5)
N1—La1—O1—C1	4.2 (6)	O4—La2—O6^iii^—La2^iii^	149.7 (2)
O11^ii^—La1—O1—C1	106.7 (6)	O4—La2—O6^iii^—C15^iii^	−77.5 (5)
O8^i^—La1—O1—C1	−68.9 (6)	O5—La2—O6^iii^—La2^iii^	23.7 (2)
O11^i^—La1—O1—C1	170.4 (6)	O5—La2—O6^iii^—C15^iii^	156.5 (5)
N2^i^—La1—O1—C1	−128.9 (6)	O6—La2—O6^iii^—La2^iii^	0.02 (17)
O1—La1—O3—La2	168.6 (3)	O6—La2—O6^iii^—C15^iii^	132.8 (6)
O1—La1—O3—C7	15.8 (5)	O7—La2—O6^iii^—La2^iii^	−73.7 (2)
O13—La1—O3—La2	23.0 (3)	O7—La2—O6^iii^—C15^iii^	59.1 (5)
O13—La1—O3—C7	−129.8 (5)	O15—La2—O6^iii^—La2^iii^	85.0 (3)
O14—La1—O3—La2	−44.7 (3)	O15—La2—O6^iii^—C15^iii^	−142.2 (6)
O14—La1—O3—C7	162.6 (5)	O16—La2—O6^iii^—La2^iii^	−38.0 (5)
N1—La1—O3—La2	167.0 (4)	O16—La2—O6^iii^—C15^iii^	94.8 (6)
N1—La1—O3—C7	14.2 (4)	O3—La2—O10^iii^—C21^iii^	104.9 (6)
O11^ii^—La1—O3—La2	86.5 (3)	O4—La2—O10^iii^—C21^iii^	57.3 (6)
O11^ii^—La1—O3—C7	−66.2 (5)	O5—La2—O10^iii^—C21^iii^	169.2 (5)
O8^i^—La1—O3—La2	−117.9 (3)	O6—La2—O10^iii^—C21^iii^	−111.4 (6)
O8^i^—La1—O3—C7	89.3 (5)	O7—La2—O10^iii^—C21^iii^	−106.1 (6)
O11^i^—La1—O3—La2	18.2 (6)	O15—La2—O10^iii^—C21^iii^	54.0 (7)
O11^i^—La1—O3—C7	−134.6 (5)	O16—La2—O10^iii^—C21^iii^	178.9 (6)
N2^i^—La1—O3—La2	−96.7 (3)	O3—La2—N3^iii^—C16^iii^	130.4 (5)
N2^i^—La1—O3—C7	110.5 (5)	O3—La2—N3^iii^—C20^iii^	−47.7 (6)
O1—La1—N1—C2	−6.0 (5)	O4—La2—N3^iii^—C16^iii^	120.7 (6)
O1—La1—N1—C6	179.1 (6)	O4—La2—N3^iii^—C20^iii^	−57.4 (5)
O3—La1—N1—C2	172.4 (5)	O5—La2—N3^iii^—C16^iii^	−14.9 (9)
O3—La1—N1—C6	−2.5 (5)	O5—La2—N3^iii^—C20^iii^	167.0 (5)
O13—La1—N1—C2	−137.2 (5)	O6—La2—N3^iii^—C16^iii^	−24.0 (6)
O13—La1—N1—C6	48.0 (6)	O6—La2—N3^iii^—C20^iii^	157.9 (5)
O14—La1—N1—C2	133.1 (5)	O7—La2—N3^iii^—C16^iii^	−87.1 (6)
O14—La1—N1—C6	−41.8 (6)	O7—La2—N3^iii^—C20^iii^	94.9 (6)
O11^ii^—La1—N1—C2	−74.7 (5)	O15—La2—N3^iii^—C16^iii^	55.5 (6)
O11^ii^—La1—N1—C6	110.5 (5)	O15—La2—N3^iii^—C20^iii^	−122.6 (5)
O8^i^—La1—N1—C2	85.5 (5)	O16—La2—N3^iii^—C16^iii^	−137.7 (5)
O8^i^—La1—N1—C6	−89.4 (5)	O16—La2—N3^iii^—C20^iii^	44.3 (6)
O11^i^—La1—N1—C2	−26.8 (6)	La1—O1—C1—O2	178.3 (6)
O11^i^—La1—N1—C6	158.3 (5)	La1—O1—C1—C2	−2.2 (9)
N2^i^—La1—N1—C2	42.9 (5)	La1—O3—C7—O4	158.7 (5)
N2^i^—La1—N1—C6	−132.0 (5)	La1—O3—C7—C6	−23.8 (8)
O1—La1—O11^ii^—C14^ii^	−126.6 (4)	La2—O3—C7—O4	−5.6 (7)
O3—La1—O11^ii^—C14^ii^	−7.4 (4)	La2—O3—C7—C6	171.9 (5)
O13—La1—O11^ii^—C14^ii^	66.1 (4)	La2—O4—C7—O3	5.8 (7)
O14—La1—O11^ii^—C14^ii^	81.8 (4)	La2—O4—C7—C6	−171.6 (5)
N1—La1—O11^ii^—C14^ii^	−67.4 (4)	La2—O5—C15—O6	−9.7 (7)
O1—La1—O8^i^—C8^i^	−79.6 (6)	La2—O5—C15—C16	166.9 (6)
O3—La1—O8^i^—C8^i^	154.8 (6)	La2—O6—C15—O5	11.0 (7)
O13—La1—O8^i^—C8^i^	87.3 (6)	La2—O6—C15—C16	−165.8 (5)
O14—La1—O8^i^—C8^i^	76.7 (6)	La2^iii^—O6—C15—O5	150.7 (5)
N1—La1—O8^i^—C8^i^	−141.9 (6)	La2^iii^—O6—C15—C16	−26.1 (8)
O1—La1—O11^i^—C14^i^	61.0 (5)	La2—O7—C8—O8	−67.8 (10)
O3—La1—O11^i^—C14^i^	−144.0 (5)	La2—O7—C8—C9	111.4 (7)
O13—La1—O11^i^—C14^i^	−149.1 (5)	La1^i^—O8—C8—O7	165.7 (5)
O14—La1—O11^i^—C14^i^	−84.5 (5)	La1^i^—O8—C8—C9	−13.5 (9)
N1—La1—O11^i^—C14^i^	79.5 (6)	La2^iii^—O10—C21—O9	150.6 (8)
O1—La1—N2^i^—C9^i^	93.4 (5)	La2^iii^—O10—C21—C20	−32.3 (9)
O1—La1—N2^i^—C13^i^	−81.9 (5)	La1^iv^—O11—C14—O12	20.2 (7)
O3—La1—N2^i^—C9^i^	−23.9 (6)	La1^iv^—O11—C14—C13	−157.4 (5)
O3—La1—N2^i^—C13^i^	160.9 (5)	La1^i^—O11—C14—O12	161.8 (5)
O13—La1—N2^i^—C9^i^	−131.2 (5)	La1^i^—O11—C14—C13	−15.8 (8)
O13—La1—N2^i^—C13^i^	53.5 (5)	La1—N1—C2—C1	7.4 (8)
O14—La1—N2^i^—C9^i^	−75.7 (5)	La1—N1—C2—C3	−173.0 (6)
O14—La1—N2^i^—C13^i^	109.1 (5)	C6—N1—C2—C1	−177.6 (6)
N1—La1—N2^i^—C9^i^	48.8 (5)	C6—N1—C2—C3	2.0 (10)
N1—La1—N2^i^—C13^i^	−126.5 (5)	La1—N1—C6—C5	173.3 (6)
O4—La2—O3—La1	−154.0 (4)	La1—N1—C6—C7	−7.1 (8)
O4—La2—O3—C7	3.0 (4)	C2—N1—C6—C5	−1.7 (11)
O5—La2—O3—La1	1.7 (3)	C2—N1—C6—C7	178.0 (6)
O5—La2—O3—C7	158.7 (4)	C13—N2—C9—C8	179.7 (6)
O6—La2—O3—La1	−14.2 (4)	C13—N2—C9—C10	1.4 (11)
O6—La2—O3—C7	142.8 (4)	La1^i^—N2—C9—C8	−4.9 (8)
O7—La2—O3—La1	88.4 (4)	La1^i^—N2—C9—C10	176.8 (6)
O7—La2—O3—C7	−114.7 (4)	C9—N2—C13—C12	−1.4 (11)
O15—La2—O3—La1	−70.3 (3)	C9—N2—C13—C14	178.9 (6)
O15—La2—O3—C7	86.7 (4)	La1^i^—N2—C13—C12	−176.8 (6)
O16—La2—O3—La1	70.2 (3)	La1^i^—N2—C13—C14	3.5 (8)
O16—La2—O3—C7	−132.8 (4)	C20—N3—C16—C15	176.8 (6)
O6^iii^—La2—O3—La1	−90.9 (4)	C20—N3—C16—C17	−0.7 (11)
O6^iii^—La2—O3—C7	66.1 (4)	La2^iii^—N3—C16—C15	−5.1 (8)
O10^iii^—La2—O3—La1	142.9 (3)	La2^iii^—N3—C16—C17	177.4 (6)
O10^iii^—La2—O3—C7	−60.1 (4)	C16—N3—C20—C19	3.4 (11)
N3^iii^—La2—O3—La1	−166.2 (3)	C16—N3—C20—C21	−175.0 (7)
N3^iii^—La2—O3—C7	−9.2 (4)	La2^iii^—N3—C20—C19	−174.8 (6)
O3—La2—O4—C7	−3.1 (4)	La2^iii^—N3—C20—C21	6.9 (9)
O5—La2—O4—C7	−31.1 (5)	O1—C1—C2—N1	−3.7 (9)
O6—La2—O4—C7	−90.1 (5)	O1—C1—C2—C3	176.6 (7)
O7—La2—O4—C7	125.0 (4)	O2—C1—C2—N1	175.9 (7)
O15—La2—O4—C7	−80.9 (4)	O2—C1—C2—C3	−3.8 (11)
O16—La2—O4—C7	42.1 (4)	N1—C2—C3—C4	0.2 (12)
O6^iii^—La2—O4—C7	−142.2 (4)	C1—C2—C3—C4	179.8 (8)
O10^iii^—La2—O4—C7	101.4 (4)	C2—C3—C4—C5	−2.8 (14)
N3^iii^—La2—O4—C7	166.0 (4)	C3—C4—C5—C6	3.2 (15)
O3—La2—O5—C15	−155.8 (4)	C4—C5—C6—N1	−1.0 (13)
O4—La2—O5—C15	−134.3 (4)	C4—C5—C6—C7	179.5 (8)
O6—La2—O5—C15	5.6 (4)	N1—C6—C7—O3	20.1 (9)
O7—La2—O5—C15	62.0 (4)	N1—C6—C7—O4	−162.4 (6)
O15—La2—O5—C15	−83.0 (4)	C5—C6—C7—O3	−160.3 (7)
O16—La2—O5—C15	126.8 (5)	C5—C6—C7—O4	17.3 (11)
O6^iii^—La2—O5—C15	−22.7 (4)	O7—C8—C9—N2	−168.0 (7)
O10^iii^—La2—O5—C15	136.9 (4)	O7—C8—C9—C10	10.2 (11)
N3^iii^—La2—O5—C15	−5.9 (8)	O8—C8—C9—N2	11.2 (10)
O3—La2—O6—C15	15.8 (4)	O8—C8—C9—C10	−170.6 (7)
O3—La2—O6—La2^iii^	−126.1 (2)	N2—C9—C10—C11	−1.3 (12)
O4—La2—O6—C15	78.5 (5)	C8—C9—C10—C11	−179.4 (8)
O4—La2—O6—La2^iii^	−63.4 (4)	C9—C10—C11—C12	1.1 (14)
O5—La2—O6—C15	−5.5 (4)	C10—C11—C12—C13	−1.1 (14)
O5—La2—O6—La2^iii^	−147.5 (3)	C11—C12—C13—N2	1.3 (13)
O7—La2—O6—C15	−125.8 (4)	C11—C12—C13—C14	−179.0 (8)
O7—La2—O6—La2^iii^	92.3 (2)	N2—C13—C14—O11	6.8 (9)
O15—La2—O6—C15	69.4 (4)	N2—C13—C14—O12	−170.8 (6)
O15—La2—O6—La2^iii^	−72.5 (2)	C12—C13—C14—O11	−172.9 (7)
O16—La2—O6—C15	−57.1 (4)	C12—C13—C14—O12	9.5 (11)
O16—La2—O6—La2^iii^	161.0 (2)	O5—C15—C16—N3	−157.5 (7)
O6^iii^—La2—O6—C15	141.9 (4)	O5—C15—C16—C17	19.9 (12)
O6^iii^—La2—O6—La2^iii^	−0.02 (18)	O6—C15—C16—N3	19.3 (9)
O10^iii^—La2—O6—C15	−120.5 (4)	O6—C15—C16—C17	−163.3 (7)
O10^iii^—La2—O6—La2^iii^	97.6 (3)	N3—C16—C17—C18	−2.6 (13)
N3^iii^—La2—O6—C15	170.0 (4)	C15—C16—C17—C18	−179.8 (8)
N3^iii^—La2—O6—La2^iii^	28.1 (3)	C16—C17—C18—C19	3.1 (14)
O3—La2—O7—C8	33.0 (7)	C17—C18—C19—C20	−0.6 (15)
O4—La2—O7—C8	−47.3 (7)	C18—C19—C20—N3	−2.7 (14)
O5—La2—O7—C8	112.3 (6)	C18—C19—C20—C21	175.5 (9)
O6—La2—O7—C8	152.1 (6)	N3—C20—C21—O9	−167.8 (9)
O15—La2—O7—C8	177.6 (6)	N3—C20—C21—O10	15.0 (10)
O16—La2—O7—C8	51.5 (6)	C19—C20—C21—O9	13.9 (14)
O6^iii^—La2—O7—C8	−147.5 (6)	C19—C20—C21—O10	−163.3 (8)
O10^iii^—La2—O7—C8	−24.8 (6)		

Symmetry codes: (i) −*x*+1, −*y*, −*z*; (ii) *x*, *y*, *z*+1; (iii) −*x*+1, −*y*+1, −*z*; (iv) *x*, *y*, *z*−1.

### Hydrogen-bond geometry (Å, °)

**Table:**

*D*—H···*A*	*D*—H	H···*A*	*D*···*A*	*D*—H···*A*
O13—H13A···O5	0.85	2.04	2.766 (8)	143
O13—H13B···O1^v^	0.85	1.91	2.721 (7)	160
O14—H14A···O5	0.97	2.23	3.062 (8)	143
O14—H14B···O9^vi^	0.83	2.44	3.225 (10)	160
O14—H14B···O10^vi^	0.83	2.22	2.712 (8)	119
O15—H15A···O7^iii^	0.85	2.14	2.948 (8)	160
O15—H15B···O12^ii^	0.85	2.16	2.849 (7)	138
O16—H16A···O8	0.85	2.12	2.760 (8)	132
O16—H16B···O17^iii^	0.85	2.05	2.843 (15)	155
O17—H17A···O9^vii^	0.82	2.13	2.816 (17)	141
O17—H17B···O9^viii^	0.82	2.39	2.758 (15)	108
O18—H18A···O2^ix^	0.82	2.31	2.75 (2)	114
O18—H18B···O18^x^	0.88	2.46	2.89 (3)	110
C11—H11···O2^xi^	0.93	2.45	3.320 (11)	155
C12—H12···O18^iv^	0.93	2.51	3.36 (2)	152

Symmetry codes: (v) −*x*+1, −*y*, −*z*+1; (vi) *x*, *y*−1, *z*; (iii) −*x*+1, −*y*+1, −*z*; (ii) *x*, *y*, *z*+1; (vii) −*x*+1, −*y*+2, −*z*; (viii) *x*−1, *y*, *z*; (ix) −*x*, −*y*, −*z*+1; (x) −*x*, −*y*+1, −*z*+1; (xi) *x*, *y*+1, *z*−1; (iv) *x*, *y*, *z*−1.
